# Shrinkage Volume, Compressive Strength, and Surface Roughness Y-TTRIA Stabilized Tetragonal Zirconia Polycrystal (Y-TZP) Using Binders Variation PVA:PEG as an Alternative Dental Implants Materials

**DOI:** 10.1055/s-0043-1761595

**Published:** 2023-03-28

**Authors:** Widaningsih Widaningsih, Vivin Ariestania, Meinar N. Ashrin, Widyasri Prananingrum, Fitri Rahmitasari, Terry Apituley, Alvin Joshua, Yeremia Alfred W., Bunga Fauzia, Chaterina D. Nanik, Oka Lestari

**Affiliations:** 1 Prosthodontic Department, Faculty of Dentistry, Hang Tuah University, Surabaya, Indonesia; 2 Dental Materials Department, Faculty of Dentistry, Hang Tuah University, Surabaya, Indonesia; 3 Faculty of Dental Medicine, Hang Tuah University, Surabaya, Indonesia; 4 Faculty of Dental Medicine, Airlangga University, Surabaya, Indonesia

**Keywords:** zirconia Y-TZP, PVA, PEG, shrinkage volume, compressive strength, surface roughness

## Abstract

**Objective** Yttria-stabilized tetragonal zirconia polycrystal (Y-TZP) is one of the materials that can be used as an alternative material for dental implants because of its good mechanical, biocompatible, and aesthetic properties. The binder used for ceramic processing to help bond is polyvinyl alcohol (PVA), which can increase the density of the ceramic material, and polyethylene glycol (PEG), which is used as a plasticizer for PVA, so it is pretty soft when pressed.

**Materials and Methods** The sample was divided into five groups for volume shrinkage and compressive strength examination consisting of K1 (PVA 100%), K2 (PEG 100%), P1 (PVA:PEG 95:5), P2 (PVA:PEG 90:10), and P3 (PVA:PEG 85:15) and four groups on the surface roughness test, namely, K (PVA:PEG 1%), P1 (PVA:PEG 2%), P2 (PVA:PEG 3%), and P3 (PVA:PEG 4%). PVA:PEG binder with various concentrations was mixed with Y-TZP. The mixture was pressed using a uniaxial pressing method and continued by sintering at 1200°C for 4 hours.

**Statistical Analysis** Least significant difference (LSD) test result showed that there was a significant difference in the compressive strength value and shrinkage volume between groups K1 and K2 and P3, and groups K2 with P1, P2, and P3. Post hoc LSD surface roughness test showed a significant difference between groups K with P2 and P3 and P1 and P3 ( *p* < 0.05). There were no significant differences ( *p* > 0.05) between K with P1 and P2 with P3.

**Results** The Y-TZP group with the PVA binder mixture had the highest compressive strength, while the highest volume shrinkage was found in the PEG group. The next highest compressive strength and volume shrinkage values were found in the PVA:PEG group with a ratio of 95:5, 102.44 MPa, and 12.5%. The best PVA:PEG ratio of 95:5 is used to make surface roughness measurement samples. The best results showed that mixing Y-TZP with 4% PVA:PEG binder had the highest surface roughness compared to other PVA:PEG binders, namely 1.3450 μm.

**Conclusion** From this study, it can be concluded that the best PVA:PEG percentage ratio to produce volume shrinkage and compressive strength is 95:5. The higher the concentration of PVA:PEG (95:5) binder mixed with Y-TZP, the higher the porosity will be.

## Introduction

The treatment for tooth loss that is currently developing is dental implants. 1 Ceramic materials were first used as metal-based implant coating materials. It is to improve the bioactive properties of the implant, namely, osseointegration and osteoconduction. The drive to meet the demand for aesthetics and metal-free implant led to the development of implant materials made of ceramics. 2 3

Zirconia (Yttria-stabilized tetragonal zirconia polycrystal ceramics or Y-TZP) is a widely used ceramic because it has good mechanical properties, including volume shrinkage and compressive strength, and surface roughness. It is a high-strength and damage-resistant biomaterial. 4 5 This material is widely used as an implant material. 6

Based on a study by Siddiqi et al, at week 12, zirconia dental implants (72.2%) in sheep mandibles had bone-implant contact, which was almost the same as titanium (60.3%). 7 This shows that the osseointegration ability of zirconia is similar to that of titanium. In addition, Y-TZP is also biocompatible because it does not release ions, so it does not cause hypersensitivity reactions, has less bacterial colonization on the surface, is corrosion resistant, and has low thermal and electrical conductivity. 3 8 According to research by Peampring and Kengtanyakich, Y-TZP in cubic form can inhibit the decrease in flexural strength compared to noncubic form 3Y-YTZP using the hydrothermal method in an autoclave for 8 hours.

One of the commonly used ways to form Y-TZP powder is by pressing method. 9 Uniaxial pressing is a relatively more straightforward process with a more affordable production price and a higher production capacity. 10 11 The addition of polymer material in the form of a binder is necessary so that the Y-TZP ceramic powder can be formed perfectly during the pressing process. 12

The binder acts as a granule binder to maintain the shape and increase the density of the ceramic after going through the sintering process. 13 Polyvinyl alcohol (PVA) and polyethylene glycol (PEG) are organic binders commonly used to form ceramics. PEG combined with PVA reduces the glass transition temperature (Tg) of PVA so that the plastic properties of ceramics can be increased. 14 15 Tg is the temperature at which a polymer changes from a rigid shape to a soft material. 16 PVA has a Tg of more than 600°C, causing PVA not to be soft enough to be pressed and needing to be combined with PEG to produce ceramics with a higher density. 17 Density changes are needed to reduce the shrinkage value. 17 According to Falqi et al, adding PEG (5 wt%) to PVA reduced PVA Tg and increased the plastic properties of the PVA-PEG mixture. 18

Shrinkage is the shrinkage of a material due to several factors, namely, variations in product thickness and irregular shrinkage during pressing. Shrinkage measurement is done to avoid volume changes in the product. Shrinkage can be seen from the sintering temperature and sintering time. Shrinkage will occur if there is a change in density from the temperature of the modeling process to room temperature. We cannot eliminate shrinkage, but it can be minimized. 19

The high density of ceramic will increase its mechanical strength, one of which is compressive strength. 20 Compressive strength is a factor that affects pressure when there is a vertical occlusion. 21 The implant material must be capable of withstanding the maximum vertical stress received by a single molar and premolar, which is 150 N. 22 If the dental implant does not have sufficient compressive strength to withstand chewing loads, a dental implant fracture may occur, causing treatment failure. 22 23 The higher the compressive strength of a material, the higher the modulus of elasticity. 24 The elastic modulus of dental implants, which is higher than the elastic modulus of bone, can cause stress shielding failing dental implant treatment. 25

Based on research by Mohanty, adding PVA binder concentrations of 2, 3, and 4% in alumina will increase the material's porosity after sintering. 13 It is due to removing the binder (binder burnout). The increase in porosity due to the loss of this binder will increase the surface roughness of a material. 8 26 The addition of PVA and PEG will produce pores after sintering. It is because the density of PVA (1.35 g/cm ^3^ ) and PEG (1.22 g/cm ^3^ ) is much lower than the density of Y-TZP (6.05 g/cm ^3^ ). 27 When porosity appears, there is an increase in surface irregularity which causes the ceramic surface to become rougher. 28 The rough and porous implants allow the implant surface area to integrate with the bone through osseointegration and osteoconduction. 29 The excellent implant properties are a moderately rough surface (Ra = 1–2 μm). 30

## Materials and Methods

The research was an original laboratory experimental research with a post-only control group research design. As much as 20 g of PEG powder is stirred in 100 mL of distilled water in a glass beaker using a spoon until it dissolves completely. Ten grams of PVA powder was mixed into 100 mL of distilled water and stirred using a magnetic stirrer on a hot plate at 80°C at 500 revolutions per minute for 2 hours.

### Shrinkage Volume

Note that 0.42 g of Y-TZP powder was mixed with 1 wt% PVA binder and 1 wt% PEG for the control group and 1 wt% PVA-PEG binder with a ratio of 95:5, 90:10, and 85:15 wt% for the treatment group with using a 3-mL syringe. Then, the mixture is stirred and dried using a mortar and pestle for 30 minutes until it becomes a dry powder. 11 Then, the powder is put into a steel mold measuring 4.5 mm wide and 25 mm long using a stainless steel spoon. The punch part of the mold is installed and then pressed with a 20-ton hydraulic press (TEKIRO) with a pressure of 150 MPa for 20 seconds. Then, the bottom of the steel mold is removed and pressed again with a 20-ton hydraulic press (TEKIRO) to remove the pressed sample. Then, the shrinkage value is recorded in the initial volume.

The pressed Y-TZP and binder mixture was sintered in a muffle furnace at 950°C for 1 hour. Then, the muffle furnace was turned off, and the Y-TZP samples were allowed to cool down to room temperature. Then, the Y-TZP sample was re-sintered in a muffle furnace at 1200°C for 4 hours. Then, the muffle furnace was turned off and cooled to room temperature. 31 Then, re-recording the shrinkage value as the final volume using a 0.001-mm digital micrometer.

### Compressive Strength

Note that 0.42 g of Y-TZP powder was mixed with 1 wt% PVA binder and 1 wt% PEG for the control group and 1 wt% PVA-PEG binder with a ratio of 95:5, 90:10, and 85:15 wt% for the treatment group, then the mixture is stirred using a mortar and pestle. The mixture was put into a steel mold with a diameter of 4 mm and a height of 100 mm, then pressed with a pressure of 150 MPa for 20 seconds. 32

The pressed Y-TZP cylinder and binder was then sintered at 950°C for 1 hour. The Y-TZP sample was allowed to cool to room temperature; then, the Y-TZP sample was re-sintered at 1200°C for 4 hours. 31 The Y-TZP powder and binder mixture was then placed into a 15 mm × 15 mm × 1.5 mm steel mold that was previously given a zinc stearate lubricant material. Punch which was the part of the mold was installed and then pressed with a 20-ton hydraulic press (TEKIRO) with a pressure of 150 MPa for 20 seconds. Then, the bottom of the steel mold was removed and repressed with 20-ton hydraulic press (TEKIRO) to release the pressing results.

### Surface Roughness

A total of 1.41 g of Y-TZP powder was mixed with 1% PVA-PEG binder at a ratio of 95:5 (wt%) for the control group, and the treatment group Y-TZP powder was mixed with 2, 3, and 4% binder PVA-PEG. The Y-TZP powder and binder mixture were then put into a 15 mm × 15 mm × 1.5 mm mold, which had previously been treated with zinc stearate lubricant. The punch, part of the mold, is installed and then pressed with a Uniaxial Pressing Machine with a pressure of 150 MPa for 20 seconds. Presenting at 950°C was carried out for 1 hour, and then cooled to 23°C in the muffle furnace. Final sintering was carried out at 1200°C for 4 hours.

The surface roughness of the Y-TZP sample was measured with a surface roughness tester (Mitutoyo). Furthermore, to see the surface morphology of the Y-TZP sample, a scanning electron microscopy (SEM) test was carried out using a HITACHI FLEXSEM 100 with 800× magnification to look at the number and pore size of the sample.

The Y-TZP sample consisted of 16 samples measuring 15 mm × 15 mm × 1.5 mm divided into four groups, namely, K (PVA-PEG binder 1%), P1 (PVA-PEG binder 2%), P2 (PVA-PEG binder 3%), and P3 (4% PVA-PEG binder).

## Result

The research data were analyzed descriptively to obtain an overview of the distribution and summary of the data to clarify the presentation of the results ( Table 1 ).

**Table 1:** The mean ± standard deviation and the Kruskal–Wallis test result of shrinkage volume, compressive strength, and surface roughness

**Shrinkage volume**				
**Groups** **(time)**	**Mean**	**Normality test** **(Shapiro–Wilk)**	**Homogeneity test** **(Levene's test)**	**ANOVA**
K1 (PVA 100%)	10.85 ± 0.7141	0.262	0.320	0.000
K2 (PEG 100%)	15.4 ± 0.9129	0.392		
P1 (PVA:PEG: 95:5)	12.5 ± 0.3948	0.139		
P2 (PVA:PEG: 90:10)	12 ± 0.8057	0.823		
P3 (PVA:PEG: 85:15)	13.3 ± 0.25	0.911		
**Compressive strength**				
**Groups** **(time)**	**Mean**	**Normality test** **(Shapiro–Wilk)**	**Homogeneity test** **(Levene's test)**	**ANOVA**
K1 (PVA 100%)	122.33 ± 26.95	0.354	0.277	0.002
K2 (PEG 100%)	62.13 ± 11.24	0.886		
P1 (PVA:PEG: 95:5)	102.44 ± 23.48	0.746		
P2 (PVA:PEG: 90:10)	97.36 ± 28.34	0.999		
P3 (PVA:PEG: 85:15)	91.56 ± 18.76	0.435		
**Surface roughness**				
**Groups** **(time)**	**Mean**	**Normality test** **(Shapiro–Wilk)**	**Homogeneity test** **(Levene's test)**	**ANOVA**
K (PVA:PEG 1%)	0.91 ± 0.110	0.765	0.455	0.019
P1 (PVA:PEG 2%)	0.93 ± 0.181	0.997		
P2 (PVA:PEG 3%)	1.255 ± 0.202	0.426		
P3 (PVA:PEG 4%)	1.345 ± 0.276	0.142		

Abbreviations: ANOVA, analysis of variance; PEG, polyethylene glycol; PVA, polyvinyl alcohol.

**Fig. 1:**
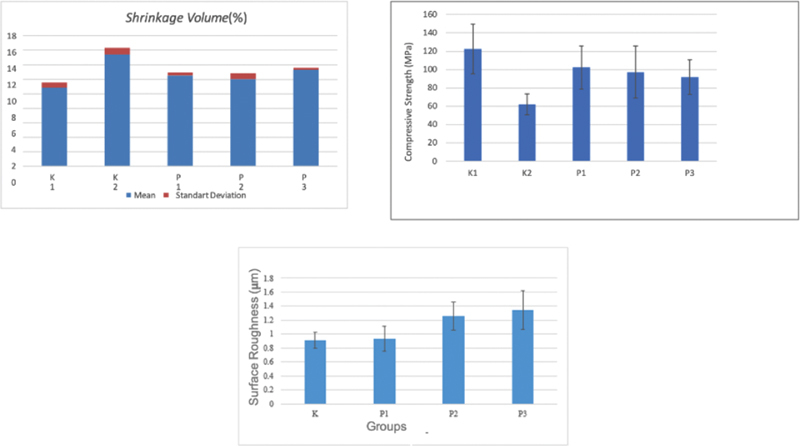
Bar chart of the mean and standard deviation of Yttria-stabilized tetragonal zirconia polycrystal (Y-TZP) shrinkage volume, compressive strength, and surface roughness with variations in the concentration of polyvinyl alcohol (PVA)-polyethylene glycol (PEG) binder.

**Fig. 2:**
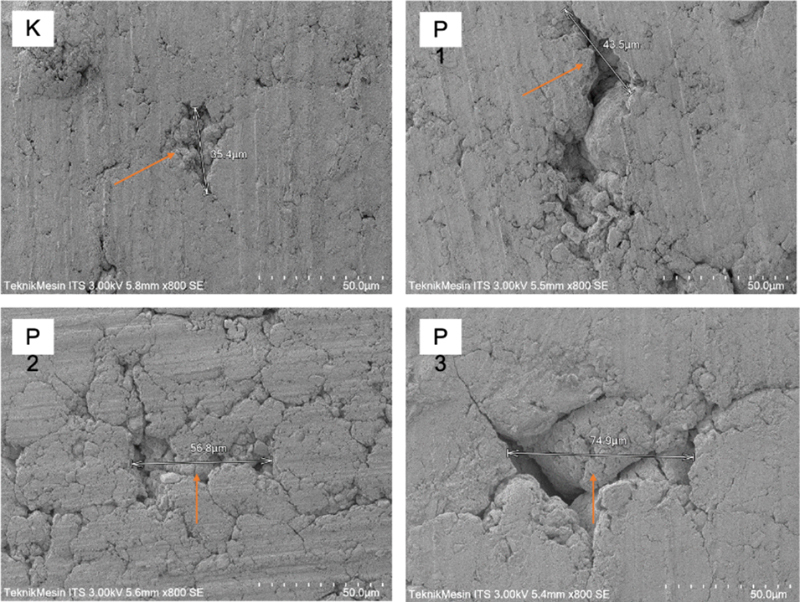
SEM (scanning electron microscopy) test results for groups K, P1, P2, and P3.

Based on Table 2 , it can be concluded that there is a significant difference in shrinkage values between groups K1 and K2, P1, P2, and P3, groups K2 and P1, P2, and P3, and groups P2 and P3. The results of the least significant difference (LSD) test showed that there was a significant difference in compressive strength values ( *p* < 0.05) between groups K1 and K2 and P3, and groups K2 and P1, P2, and P3. The post hoc LSD surface roughness showed a significant difference between groups K with P2 and P3 and P1 and P3 ( *p* < 0.05). There was no significant difference between K and P1 and P2 and P3 ( *p* > 0.05) ( Figs. 1 and 2 ).

**Table 2:** LSD test shrinkage volume, compressive strength, and surface roughness

**Shrinkage volume**					
**Groups**	**K1**	**K2**	**P1**	**P2**	**P3**
K1		0.000 ^a^	0.003 ^a^	0.025 ^a^	0.000 ^a^
K2			0.000 ^a^	0.000 ^a^	0.001 ^a^
P1				0.304	0.091
P2					0.012 ^a^
P3					
**Compressive strength**					
**Groups**	K **1**	**K2**	P **1**	**P2**	**P3**
**K1**		0.000 ^a^	0.143	0.069	0.027 ^a^
**K2**			0.005 ^a^	0.012 ^a^	0.033 ^a^
**P1**				0.700	0.413
**P2**					0.661
**P3**					
**Surface roughness**					
Groups	**P1**	P2	**P3**		
**K**	0.891	0.032 a	0.010 a		
**P1**		0.042 a	0.013 a		
**P2**			0.540		

Abbreviation: LSD, least significant difference.

a *p* < 0.05 = significant difference.

SEM test results showed that the higher the binder concentration, the number and size of the pores would increase.

## Discussion

Shrinkage is the shrinkage of a material due to several factors, namely, variations in product thickness and irregular shrinkage during pressing. Shrinkage measurement is done to avoid shrinkage of the product. 19 The shrinkage value of a material indicates the high modulus of elasticity of the material so that the material for making dental implants is strong against impact or pressure, which can cause fracture. 33

There was a significant difference between groups K1 and K2, P1, P2, and P3 due to the effect of the PVA binder added to the K1 group, which was greater than the groups K2, P1, P2, and P3. The PVA binder functions as an adhesive so that the ceramic can maintain its shape during sintering so that changes in the volume of the K1 group samples before and after sintering give a low shrinkage value.

In the group with pure PEG and PEG, the ratio was more significant than PVA, causing the volume shrinkage value to be higher. PEG has a low Tg, whereas a polymer with a low Tg (more elastic chains) has a lower density. 12 13 Using too much binder can decrease the density of material so that the result becomes easy to shrink when sintering. 17

The treatment groups P1, P2, and P3 were treated with a mixture of PVA-PEG binders. Mixing these two binders binds the ceramic powder together so that the ceramic can maintain its shape during the sintering process. The combination of PEG and PVA can give ceramics plastic properties by reducing the properties of PVA, namely, the Tg. *Plasticity* is required for ceramic materials to have a higher density. 18 In group P1, a shrinkage value of 12.5% was obtained; this was because group P1 was given a PVA-PEG binder with a ratio of 95:5. The P1 group has a higher mechanical strength than the P2 group. So the sample in group P1 has a larger shrinkage volume when compared to group P2.

In the compressive strength examination, there was a significant difference between the K1 and K2 and P3 groups due to the effect of the PEG binder added to the PVA-PEG mixture, causing the mechanical strength of the Y-TZP ceramic to decrease. Based on research conducted by Pigram and Freer (1994), 38 it takes PEG with twice the concentration of PVA to achieve the same mechanical strength.

There was no significant difference between the K1 and P1 and P2 groups because the total PEG ratio in the P1 and P2 groups had not been able to produce the desired plastic properties. Based on research conducted by Falqi et al, the PVA-PEG mixture in groups P1 and P2 still have a Tg of 53.85°C and 54.39°C, so it is not plastic enough at room temperature. 18

There was a significant difference between the K2 and the P1, P2, and P3 groups. This significant difference is due to the large PEG ratio difference in K2 compared to P1, P2, and P3. The higher the amount of PEG in the PVA-PEG binder, the mechanical ability of the ceramic will decrease.

There was no significant difference between the P1 group and the P2 and P3 groups, indicating the lack of influence of PEG adhesion bonds in breaking the hydrogen bonds, which caused a decrease in PVA Tg so that PVA was not plastic enough to do uniaxial pressing. 23

The Y-TZP sample in all control and treatment groups already has sufficient compressive strength to withstand the vertical stress generated during the mastication process, which is 150 N. 23 Even though the stress generated during mastication is smaller than the compressive strength of the Y-TZP sample, this repeated stress can cause deformation of the Y-TZP sample. This deformation can lead to uneven stress distribution and dental implant failure. 24

The compressive strength of a material also affects its elastic modulus. The higher the compressive strength of a material increases, the elastic modulus will also increase. 24 The elastic modulus of dental implant material, higher than the human bone, can cause stress shielding failing dental implant treatment. 24 The Y-TZP sample in group K1 has the highest compressive strength (122.22 MPa) compared to the other groups. However, Y-TZP in P1 could not reach the compressive strength of human cortical bone, which was 200 MPa, 34 35 so it did not meet the requirements as a base material for dental implants. The larger the size of the Y-TZP powder, the lower the mechanical strength. 36

The surface roughness test ( Table 1 ) shows that adding PVA-PEG concentrations of 1, 2, 3, and 4% will increase the surface roughness of Y-TZP. It happens because higher binder concentration will increase the porosity of a material after the sintering process. 9 The increase in porosity in the material occurs because more and more binder is lost during the sintering process at high temperatures, and the surface will become relatively rougher. 27

The results of statistical analysis using the LSD post hoc test showed that there were significant differences in surface roughness in groups K and P2, K and P3, P1 and P2, and P1 and P3. It was because there were more binder removal processes in groups P2 and P3 compared to groups K and P1. During the mixing process of PVA and PEG binders, hydrogen bonds form between the two macromolecules. 18 PVA and PEG binders, through their –OH groups, will also form hydrogen bonds with Y-TZP. 37 The sintering stage aims to remove the binder. Binder must remove to avoid defects such as black discoloration, closed porosity, bloating, and defects in the final shape of the ceramic. 13 In ceramics, sintering is carried out at 1200°C. Binder removal usually occurs at 600 to 700°C for pure polymers and can be higher for ceramic powders. Binder decomposition begins at 200to 400°C where the C-H and C = O bonds disappear, while the C = C bond increases at the same temperature. The C = C bond will decrease significantly at 500°C and slow down at 800°C. Loss of binder bonds will cause pores to appear on the sample's surface and reduce the sample's density. 28

The smaller the density of the material, the larger the pore size produced after sintering. According to research by Zare et al, adding 5wt% PEG will produce huge pores after sintering. 27 It is because the density of PEG (1.22 g/cm ^3^ ) is much lower than the density of Y-TZP (6.05 g/cm ^3^ ). The addition of 95 wt% PVA, which has a density of 1.35 g/cm ^3^ , will also reduce the density of Y-TZP by producing smaller and more pores than 5 wt% PEG. When porosity appears as a result of the removal of the binder, there is an increase in surface irregularity, causing the ceramic surface to become rougher. 29 Based on Table 1 , the average surface roughness in groups P2 and P3 is 1.2550 and 1.3450 µm. This follows the criteria for good implant surface roughness which is 1 to 2 µm. 31

Table 2 shows that there are insignificant differences in surface roughness values between groups K and P1 and between groups P2 and P3. It is because PEG has higher thermal stability when compared to PVA. PEG decomposition begins above 330°C while PVA decomposition begins at 240°C. 18 Polymer decomposition continues at temperatures higher than 500°C; thermogravimetric analysis data shows no decrease in sample weight above 500°C. It is reported that a low carbon residue remains in the ceramic sample after sintering at high temperatures. 27

Based on the results of the SEM test, it was seen that the average pore size for each group was 13.9 µm (K), 21.3 µm (P1), 47.3 µm (P2), and 53.6 µm (P3). The higher the concentration of PVA and PEG binders, the number and size of the pores will increase. It is directly proportional to the surface roughness, where the higher the binder concentration, the more surface roughness will increase.

## Conclusion

From the results of this study, it can be concluded that the comparison of the use of PVA:PEG binder with a ratio of 95:5 to zirconia Y-TZP material has high compressive strength and low volume shrinkage, the greater the ratio of PEG, the compressive strength of Y-TZP and shrinkage volume of Y-TZP will also increase. The higher the concentration of PVA:PEG, the higher the pore size and porosity of Y-TZP.
